# Genome Sequence of *Pantoea ananatis* Strain CFH 7-1, Which Is Associated with a Vector-Borne Cotton Fruit Disease

**DOI:** 10.1128/genomeA.01029-15

**Published:** 2015-09-10

**Authors:** Enrique G. Medrano, Alois A. Bell

**Affiliations:** Insect Control and Cotton Disease Research Unit, U.S. Department of Agriculture, Agricultural Research Service, College Station, Texas, USA

## Abstract

*Pantoea ananatis* is a bacterium with versatile niches that vary from pathogenic to beneficial. We present the genome of strain CFH 7-1, which was recovered from a diseased greenhouse cotton boll previously caged with a field-collected cotton fleahopper (*Pseudatomoscelis seriatus*). These data will assist in deciphering the infection process.

## GENOME ANNOUNCEMENT

Representatives of *Pantoea ananatis* are motile, Gram-negative rods that have been isolated from numerous environments; strains include both human (1, 2) and plant pathogens (3), endophytes (4), and biocontrol agents (5). In agricultural production, *P. ananatis* is regarded as an emerging pathogen since its host range continues to expand seasonally, hindering economic gains. Cotton is a major economic commodity grown in regions throughout the world. Vector-borne diseases have a substantial negative impact on productivity (6). The cotton fleahopper (*Pseudatomoscelis seriatus*) is an established and important cotton pest. Strain CFH 7-1 was isolated from a diseased greenhouse cotton boll that was first caged with a cotton fleahopper collected from an infested field plot. Infectivity using Koch’s postulates confirmed the opportunistic disease capacity of CFH 7-1. The CFH 7-1 whole-genome sequence project will advance the process of detecting genes implicated in the disease paradigm.

A high-quality draft whole-genome sequence (38-fold coverage) of strain CFH 7-1 was generated using a Roche 454 GS Junior system . Three shotgun library runs were conducted, resulting in over 131 Mb summated from 46.5 Mb (105,362 reads; average length 442 bases), 57.0 Mb (128,827 reads; average length 442 bases), and 27.1 Mb (297,758 reads; average length 703 bases). Employing the same genomic DNA stock, 3- and 8-kb paired-end Titanium libraries were constructed and pyrosequenced, producing 37 Mb (165,540 reads with 19,292 paired reads) and 34 Mb (155,367 reads with 17,448 paired reads), respectively. The genome was constructed using the GS *de novo* assembler 454 version 2.7 and the CLC Genomics Workbench Linux platform, resulting in scaffolds equaling 5.2 Mb. Scaffold gaps were sealed by cloning PCR products and Sanger sequencing by means of an ABI PRISM 3100 genetic analyzer. Putative coding sequences were identified with the NCBI BLAST program for manual annotation and computationally utilizing the Prokaryotic Genome Annotation Pipeline program at the NCBI; both sets of results were manually curated.

A total of 4,073 coding sequences (CDSs) were predicted, along with 6 rRNA operons and 53 tRNAs. The GC content of the chromosome (4.2 Mb) was 53%. Two plasmids were identified, and their GC content was 52% for the larger plasmid (0.29 Mb) and 49% for the smaller plasmid (0.14 Mb). Components of a type VI secretion system were identified, and this delivery machine is known to be involved in plant infection (7). Additionally, a putative gene that encodes for a protein involved in fusaric acid resistance was revealed. Collectively, these data will assist in dissecting the cotton infection process and bacterial methods used to compete in the environment with other microbes. These data provide a significant advancement in new knowledge of a species that consists of strains with diverse habitats.

### Nucleotide sequence accession numbers. 

This whole-genome shotgun project has been deposited at DDBJ/EMBL/GenBank under the accession number LFLX00000000. The version described in this paper is the first version, LFLX00000000.1.
